# Underlying Mechanism of Aconitum Lizhong Acting on Experimental Hypothermia with Indigestion in Rats: Role of Ghrelin

**DOI:** 10.1155/2012/542461

**Published:** 2012-07-31

**Authors:** Xin Zhao, Yong Wang, Shijun Yang, Wentong Zhang, Chengzhe Zu, Taotao Zhang, Binghua Tang, Baochun Zhang, Guozhang Li, Bo Deng, Dayong Cai

**Affiliations:** ^1^Center of Pharmacology and Toxicology, Institute of Medicinal Plant Development, Chinese Academy of Medical Sciences, Peking Union Medical College, Beijing 100193, China; ^2^School of Basic Medicine, Beijing University of Chinese Medicine, Beijing 100029, China

## Abstract

This study is aimed to investigate the Aconitum Lizhong pill (ALZ) pharmacological actions on hypothermia with indigestion, especially the ghrelin roles. The littermate-matched rats were randomly divided into four groups. Control did sham operation or standard diet, Model carried out interscapular brown adipose (IBA) removal with standard diet, Fat-diet did IBA removal with fat-diet, and ALZ did IBA removal and fat-diet with 4.536 g/kg/d ALZ. The potency of adaptive thermogenesis, ghrelin levels in plasma or gastric mucosa, thyroid hormones and metabolite in sera, expression of ghrelin mRNA, and protein in gastric mucous membrane were determined. ALZ relieved the hypothermia processes with indigestion, via inhibiting ghrelin expression and increasing ghrelin secretion; the dynamics from the therapy is supported with the energy changes as less body weight loss, less plasma lipid decrease, more plasma *T*_3_ or *T*_4_ increase with TSH decrease, and more compensation of thermogenic AUC decrease. Ghrelin played key roles in the actions of ALZ on the hypothermia with indigestion. The pharmacological mechanisms of ALZ involved the homeostasis of ghrelin expression and secretion.

## 1. Introduction

Aconitum Lizhong pill (ALZ) is a classical herbal product for the patients with Spleen Yang Deficiency in traditional Chinese medicine (TCM) [1]. This syndrome is clinically characterized with cold body and limbs, diarrhea, bad appetite, and lassitude [2]. The features are related to the hypothermia with indigestion. ALZ composes of five dried medical herbs, effects on the thermogenesis and digestion [3]. 

Ghrelin, a peptide hormone mainly secreted from gastric mucosa, regulates food intake, energy balance, and gut motility [4, 5]. The disorders of gut motilities and energy metabolisms always result in chronic gastrointestinal functional disturbances [6], which are matched with the ALZ indications [7]. Our data have previously demonstrated that the interscapular brown adipose (IBA) removal decreases adaptive thermogenesis [8], this hypothermia involved the energy charge in hepatocytes [9], skeletal muscles [10], and skeletal myosin ATPase activity [11]. ALZ could moderate these changes [8–11]. 

In the present study, ghrelin is supposed to be a bridging signal between both processes, as the key effecter of ALZ actions for Spleen Yang Deficiency. Matched with rat littermates, IBA tissue is removed to induce hypothermia, high fatty diet was fed to replicate indigestion, and ALZ was administered intragastrically to moderate the processes of the syndrome [8–11]. The effect of ALZ was observed as lipid metabolism, thyroid hormones, and adaptive thermogenesis, and its pharmacological mechanisms were further explored as ghrelin expression and secretion.

## 2. Materials and Methods

### 2.1. Animals and Materials

Wistar rats (18 pairs of male and female) weighing 200–220 g were supplied by the Animal Centre of the Chinese Academy of Medical Sciences. Standard rodent pellets or Fat-diet pellets (83% basic feedstuff, 15% triglyceride, and 2% cholesterol) for rats were prepared by Beijing Scientific Animal Feedstuff Company. Rats were housed in a SPF laboratory. All experiment procedures were performed in accordance with the guidelines of animal experiments from the Committee of Medical Ethics, National Health Department of China. 

Aconitum Lizhong pill is a thermogenesis-promoting prescription derived from TCM, consisting of five dried medical herbs (2 : 4 : 3 : 2 : 2 w/w) with representative molecules, such as *Aconitum carmichaelii* with aconitine, *Codonopsis tangshen* or *Atractylodes macrocephala* with atractylenolide III, Zingiber officinale with gingerol, and Glycyrrhiza uralensis with glycyrrhetic acid. According to Pharmacopoeia of the People's Republic of China (2010), the decoction was prepared and concentrated to 0.4536 (g/mL) by the pharmaceutical department, Beijing University of Chinese Medicine.

### 2.2. Experimental Design

**Figure 1:**
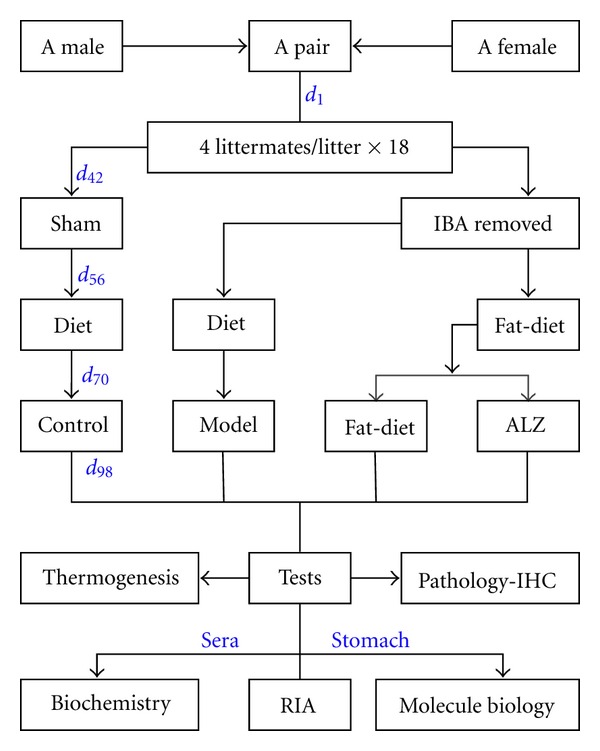
Rat approached in four groups. IBA: interscapular brown adipose; ALZ: Aconitum Lizhong pill; IHC: immunohistochemistry; RIA: radioimmunoassay.

The rats were randomly divided into 18 pairs with one male and one female in a cage (D650 cm × W450 cm × H200 cm). The male rat was taken out of the cage after the vaginal plug was found. The four littermates (8.12 ± 1.83 g) per litter were bred and randomly divided into four groups. With 18 litters, the weight was not significantly different among the groups (Figure 1).

**(1) Control**

Rats underwent sham operation to remove brown adipose tissue at *d*_42_ after birth, fed with standard rodent pellets continuously from *d*_56_, and administrated intragastrically with saline (10 mL/kg) from *d*_70_. After anesthesia with anesthetic ether, rat thermogenesis was observed, and the plasma or gastric tissues were harvested at *d*_98_.

**(2) Model**

Rats underwent surgery to remove brown adipose tissue at *d*_42_ after birth; the others were the same as the Control group.

**(3) Fat-Diet**

Rats were fed with fat-diet from *d*_56_; the others were the same as the Model group.

**(4) ALZ**

Rats were administrated intragastrically with ALZ (4.536 g/kg) from *d*_70_ to *d*_98_; the others were the same as the Fat-diet group.

### 2.3. Surgical Procedures

IBA was removed at *d*_42_ after birth from all rats except Control rats. Briefly, rats were anesthetized with an intraperitoneal injection of sodium pentobarbital (50 mg/kg). The IBA was exposed through an incision from neck to the middle of the back with aid of binocular stereomicroscopy. A butterfly-shaped IBA was revealed and dissected with mechanical arms of stereomicroscope. Vessels and nerves were cut with electrothermal knife edge. The skin incision was sutured with some subcutaneous tissue.

### 2.4. General Characteristics of Rats

**(1) Growth Ratio**

After fasting for 12 h, each rat was weighed at *d*_42_ (*W*_42_) and *d*_98_ (*W*_98_). The growth ratio was calculated from the formula *G*_*r*_ = (*W*_98_ − *W*_42_) ÷ *W*_42_ × 100%.

**(2) Plasma Biochemistry**

Blood samples were taken after thermogenesis at *d*_98_. The plasma was separated by centrifugation (1600 ×g for 15 min) and stored at −70°C. Levels of total protein (TP), total cholesterol (TC), triglycerides (TG), and glucose (Glu) in plasma were determined by the use of an AutoAnalyzer (7060, Hitachi, Japan).

### 2.5. Nonshivering Thermogenesis

Thermogenesis induced with isoproterenol was evaluated in thermoneutrality (28°C) at *d*_42_, *d*_70_, and *d*_98_. Briefly, the rats were under ether anesthesia. Three thermometers were used to measure different sites of temperatures. The first one was fixed in the colon for core temperature, the second one was fixed on the skin of the left leg for skin temperature, and the third one was set in the air of the laboratory for controlled thermoneutral temperature. The thermometers were connected with PowerLab. Thirty minutes after rats awoke from anesthetization, the temperatures were recorded, isoproterenol hydrochloride (106 *μ*g/kg, Sigma, USA) was given to rats through venepuncture in the tail vein, then the temperatures were recorded for 120 minutes continuously [8]. A curve was made from the duration of 120 minutes and the core temperatures with GraphPad Prism 4, thermogenic peak, and AUC were read as the peak values (°C) and the area under curve (°C·h) in 120 minutes.

### 2.6. Radioimmunoassay (RIA)

**(1) Plasma RIA**

Blood sample of each rat was collected. The blood was centrifuged (1600 g × 15 min) at 4°C, and the plasma supernatant was transferred into eppendorf tubes and stored at −80°C. The plasma levels of total ghrelin and the thyroid-related hormones, including total triiodothyronine (*T*_3_), total thyroid hormone (*T*_4_), or thyroid stimulating hormone (TSH), were detected on radioimmunoassay according to the manufacturer's instructions of RIA Kit, reactivity with rat ghrelin 100%. The radioactivities were counted using a Gamma Counter (Auto-Gamma Counting Systems; PACK-ARD Instrument, Meriden, USA). The within-and-between assay coefficients of variation for the ghrelin assay were both less than 5% (Phoenix Biotech, Beijing, China). Each rat sample was assayed, and a standard curve was obtained from measurements in duplicate. The content of ghrelin was calculated as pg/mL of plasma.

**(2) Gastric Mucus RIA**

Chopped tissues were boiled for 10 min in l mol/L acetic acid and homogenized at 4°C. The extract solution was centrifuged (24,000 g × 30 min). The tissue extract solution was loaded onto a Sep-Pak C18 cartridge and was preequilibrated with 0.5 m mol/L acetic acid, and the adsorbed material was eluted with 4 mL 50% CH_3_CN containing 0.1% trifluoroacetic acid. After the samples were lyophilized, the residue was dissolved in radioimmunoassay buffer and assayed according to the manufacturer's instructions. The level of ghrelin was calculated and expressed as *μ*g/mg of total protein.

### 2.7. Immunohistochemistry (IHC)

The immunohistochemical localization of ghrelin was detected according to the method described by Li et al. [12] with some modification. After the tissue sections (6 *μ*m) were treated with 3% normal goat serum for 1 h at 25°C, then rabbit polyclonal antibody (Santa Cruz) against rat ghrelin was applied to the gastric sections (1 : 200) and incubated overnight. An avidin-biotin peroxidase kit was used according to the manufacturer's instructions (Boster, Wuhan, China). One set with hematoxylin counterstaining was used for cellular location. Another set without counterstaining was used for morphometry. The mean optical density (OD), positive area (AP), and observed area (AT) of ghrelin IHC were determined with Image Pro-Plus 7.0.1 (Bethesda, USA) under 40 magnifications. The total existing ghrelin was calculated by the formula OD × (AP ÷ AT)^3/2^. 

### 2.8. Quantitative RT-PCR for Ghrelin mRNA

Total RNA was extracted from the gastric mucosa using TRIzol according to the manufacturer's instructions (Promega). Trace contamination of DNA was removed by DNase digestion (Promega). The cDNA was synthesized from 2.0 *μ*g total RNA using SuperScript III Reverse Transcriptase (Invitrogen). The following primers were designed to amplify rat ghrelin (sense primer, CAG GTT CCA GCT TCT TGA; antisense primer, GAC AGC TTG ATG CCA ACA; 191 bp) and *β*-actin (sense primer, CAC CCG CGA GTA CAA CCT TC; antisense primer, CCC ATA CCC ACC ATC ACA CC; 207 bp). Real-time quantitative PCR was performed using TaKaRa SYBR Premix Ex TaqTM according to the manufacturer's instructions. Amplification reactions were performed using an IQ 5.0 ABI 7500 (Biorad, USA). Initial template denaturation was performed for 30 s at 95°C. The cycle profiles were programmed as follows: 5 s at 95°C (denaturation) and 34 s at 60°C (annealing and extension). Forty cycles of the profile were run and the final cooling step was continued for 30 s at 4.0°C. Quantitative measurement of ghrelin mRNA was achieved by establishing a linear amplification curve from serial dilutions. Amplicon size and specificity were confirmed by the melting curve analysis (2^−ΔΔCT^ as the mode) and the 2% agarose gel electrophoresis from the other amplification designed with both of 28 cycles and final extension was 72°C for 10 min.

### 2.9. Western Blot Analysis for Ghrelin Protein

Gastric corpus mucosa was lysed in a solution containing 10 mM potassium phosphate (pH 6.8), 1 mM EDTA, 10 mM 3-[(3-cholamidopropyl)dimethylammonio]-1-propanesulfonate (CHAPS, GE Healthcare Life Sciences, Beijing, China) and protease inhibitor cocktail Tablet Complete (Roche Diagnostics). The homogenate was then centrifuged at 12,000 g for 30 min at 4°C. The protein concentrations of supernatant were determined with the Bradford method. Equal amounts of protein (20 *μ*g) were diluted in SDS buffer, heated at 98°C for 10 min, and loaded into a 10% to 20% Tricine gradient gel. After SDS-PAGE, proteins were transferred to nitrocellulose membranes (MSI, Westboro, MA, USA) for 6 h at 4°C and then incubated with rabbit antibody against rat ghrelin (1 : 200, Santa Cruz Biotechnology). The membranes were then incubated in a secondary alkaline phosphatase conjugated goat anti-rabbit IgG antibody (1 : 5000, Santa Cruz Biotechnology) for 1 h at room temperature. *β*-actin (Santa Cruz Biotechnology) was used as an internal control for protein examined. Protein bands were visualized by enhanced chemiluminescence (ECL) system (GE Healthcare Life Sciences, Beijing, China). Images were captured by a ChemiDoc XRS cooled charge-coupled device camera and analyzed with Quantity One software (Bio-Rad Laboratories, Hercules, CA, USA).

### 2.10. Statistical Analysis

All data were standardized with data for rat partners in the same litter to control the variations due to heredity among the various litters. Make the average of 4 littermates in each litter equal to the total average of all rats in 18 letters (*N* = 72), excepting for biological molecule tests (ghrelin mRNA and protein) in 7 letters (*N* = 28).

Results were expressed as mean ± SD for each group. Comparisons were performed with one-way ANOVA; ghrelin levels were linear regressed with thermogenic AUC potency. A *P* < 0.05 was considered statistically significant.

## 3. Results

### 3.1. General Characters of the Rats

#### 3.1.1. Growth Ratio in the Rats

Compared with Control (Table 1), the growth ratio from *d*_42_ to *d*_98_ decreased significantly in Model and Fat-diet (*P* < 0.01). ALZ could slow down this decrease tendency (*P* < 0.01). 

#### 3.1.2. Plasma Biochemistry in the Rats

Compared with Control (Table 2), levels of TP, TC, TG, and Glu decreased significantly in Model and Fat-diet (*P* < 0.01). ALZ could reverse the changes of TC, TG, and Glu (*P* < 0.01), but level of TP decreased (*P* < 0.01). 

#### 3.1.3. Thyroid-Related Hormones in the Rats

The level of *T*_3_ was highest in Model (*P* < 0.01) and significantly reduced in Fat-diet and ALZ (*P* < 0.01). So did the similar changes of *T*_4_ and the reverse changes of TSH (Table 3).

### 3.2. Thermogenesis in the Rats

Isoproterenol-induced thermogenesis (Table 4) was demonstrated in each group at *d*_42_, *d*_70_, and *d*_98_. The thermogenic peak at *d*_42_ had no significant difference between each group. At *d*_70_, it decreased significantly in Model and Fat-diet (*P* < 0.01), and ALZ did not relive the hypothermia. At *d*_98_, ALZ promoted thermogenesis significantly (*P* < 0.01), so did the thermogenic AUC (Table 5). 

### 3.3. Ghrelin in the Rats

**(1) Plasma Ghrelin Level**

Rats in Model (*P* < 0.01) and Fat-diet (*P* < 0.05) showed significant increment of plasma ghrelin level compared with Control (Table 6). ALZ increased ghrelin secretion compared with Fat-diet (*P* < 0.05).

**(2) Gastric Ghrelin Level**

The gastric ghrelin (Table 6) detected by RIA decreased significantly in Model and Fat-diet (*P* < 0.01). ALZ moderated this potency (*P* < 0.01).

**(3) Ghrelin Cellular Localization**

**Figure 2:**
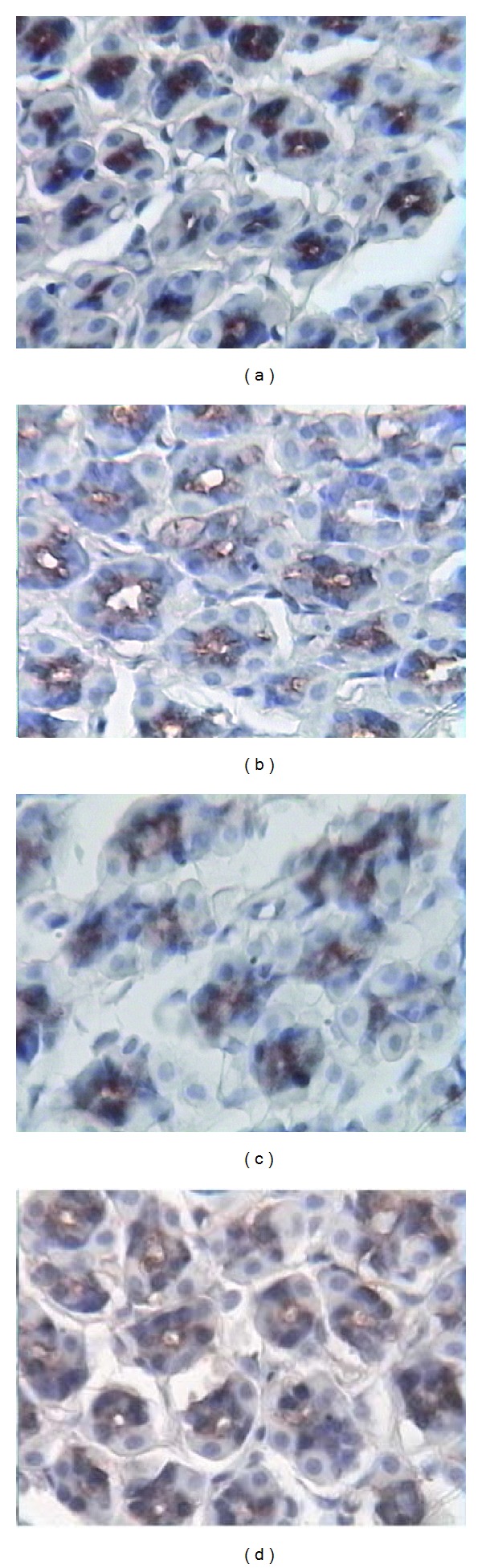
Ghrelin cellular location in fundic gland (IHC × 400). (a) Control rats, (b) Model rats, (c) Fat-diet rats, (d) ALZ rats.

In Control rats, the positive staining was located in the cytoplasm of typical gastric endocrine cells; positive-stained cells in the stomach were found from the neck to the base of the oxyntic gland (Figure 2(a)). In Model, the positive staining was located in both of the cytoplasm and the lumen of pyloric gland (Figure 2(b)). Compared with Model, Fat-diet rats showed stronger gastric ghrelin staining (Figure 2(c)). Compared with Fat-diet rats, ALZ (Figure 2(d)) showed stronger gastric ghrelin staining.

**(4) Existed Gastric Ghrelin**

The amount of existed ghrelin in gastric mucosa (Table 6) reduced significantly in Model and Fat-diet (*P* < 0.01). ALZ increased that significantly.

**(5) Gastric Ghrelin mRNA**

**Figure 3:**
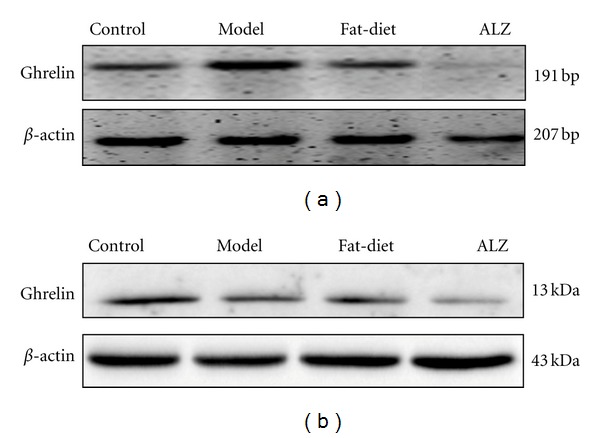
Ghrelin expression in gastric mucosa. (a) Ghrelin mRNA expression was assessed by RT-PCR, and *β*-actin was used as control, (b) Ghrelin protein was assessed by western blot using antighrelin, and *β*-actin was used as control. Ghrelin band intensity was normalized to *β*-actin band intensity. The images were representative of each littermate of four groups originated from the same litter.

The levels of ghrelin mRNA (Table 6, Figure 3(a)) were higher in Model and Fat-diet than Control (*P* < 0.01). ALZ regulated down its expression (*P* < 0.01).

**(6) Gastric Ghrelin Protein**

Compared with Control (Table 6, Figure 3(b)), the amount of ghrelin protein in gastric mucosa increased in Model and Fat-diet (*P* < 0.01). ALZ could reverse the changes (*P* < 0.01).

**(7) Ghrelin Correlated with Thermogenesis**

An inverse relation was found between plasma ghrelin content and existing gastric ghrelin amount in all rats (*r* = 0.664, *P* < 0.001, *N* = 72). In addition, an inverse relation was found between the plasma ghrelin content and the thermogenic AUC (*r* = 0.539, *P* < 0.001, *N* = 72). The positive linear relation was found between gastric ghrelin level and the thermogenic AUC (*r* = 0.672, *P* < 0.001, *N* = 72).

## 4. Discussion

ALZ is one of the classical prescriptions for Spleen Yang Deficiency in TCM [13]. The syndrome had involved the hypothermia with indigestion [14]. The hypothermia-like symptoms are characterized with cold limbs, weakness, lassitude, pale tongue, and weak pulse [1, 14]. The indigestion like symptoms did as cold stomach ache, vomiting, diarrhea, indigestive stool, anorexia, white fur, and thread deep pulse [1, 14]. Clinical research revealed that ALZ had a variety of therapeutic effects such as warming stomach, dispelling cold, relieving vomiting, and diarrhea [14]. Meanwhile, pharmacological research reveals that ALZ is effective for promoting thermogenesis and adjusting gastrointestinal functions [13]. Ghrelin increases food intake and decreases energy expenditure, leading to a positive energy balance [15]. There was a potential use of ghrelin for gastrointestinal diseases [12] and temperature regulation [16]. The pharmacologic actions of ALZ involved energy charges [9, 10] and ATPase activity [11]. The directive molecules of adaptive thermogenesis were the uncoupling proteins in brown adipocytes, hepatocytes, striated muscles, gonocytes, and neurons [17]. According to the principles in multilayer network for drug-NEI-disease [18], ALZ was hypothesized to increase ghrelin secretion, as one of its effectors, consequently to relieve the pathogenesis of hypothermia with indigestion.

In the present study, the hypothermia was induced by IBA removal, and the indigestion was replicated with fat-diet. The hypothermia with indigestion was significantly relived with ALZ [8]. The results showed the following. (1) Compared with Control rats, IBA removal caused less weight, lower plasma metabolites (TP, TC, TG, and Glu), higher thyroid hormones (*T*_3_, *T*_4_) with lower TSH, lower thermogenic peak or AUC, higher gastric ghrelin expression, less gastric ghrelin detected by IHC or IRA, and more plasma ghrelin. It suggested that IBA removal may upregulate ghrelin expression and ghrelin secretion. (2) Compared with Model rats, Fat-diet rats were characterized with further less weight, higher lipid metabolites (TP, TC, and TG), lower thyroid hormones (*T*_3_, *T*_4_) with higher TSH, higher thermogenic peak or AUC, lower gastric ghrelin expression, more gastric ghrelin content detected by IHC, less gastric, and plasma ghrelin level detected by IRA. It suggested that Fat-diet relieves the upregulation of ghrelin secretion, decreases acute cold tolerance, and increases chronic cold tolerance. (3) Compared with Fat-diet rats, ALZ-administrated rats were characterized with higher weight, higher plasma metabolites (TC, TG, and Glu), lower thyroid hormones (*T*_3_, *T*_4_) with further higher TSH, higher thermogenic peak or AUC, lower gastric ghrelin expression, more gastric ghrelin content detected by IHC, higher gastric, and plasma ghrelin level detected by IRA. It suggested that ALZ downregulates ghrelin expression, promotes ghrelin secretion, and consequently increases cold tolerance. ALZ made an economical energy balance for maintaining a stable cold tolerance in the model of hypothermia with indigestion. 

**Figure 4:**
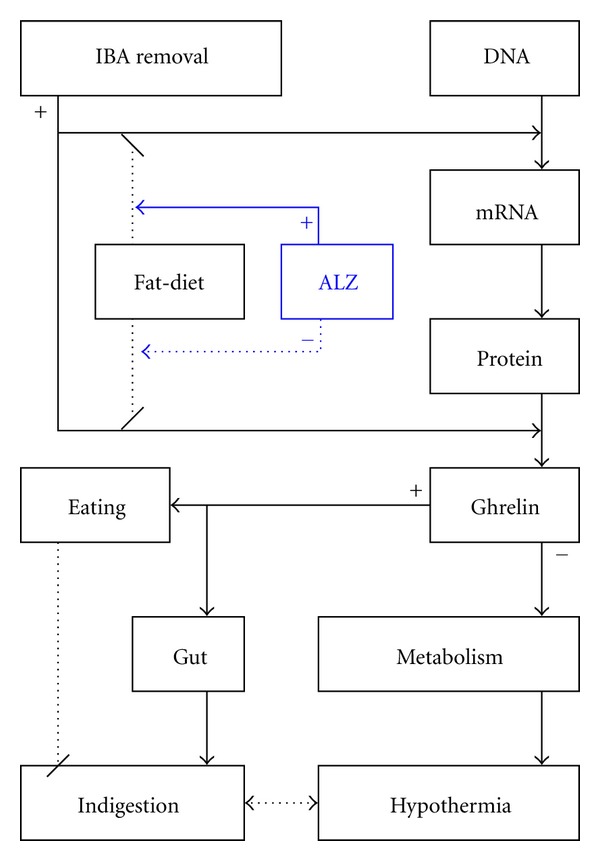
Ghrelin role in ALZ for the hypothermia with indigestion.

The ghrelin secretion is close to energy balance and thermogenesis (Figure 4) [19]. Plasma ghrelin elevation could inhibit metabolism, increase food intake, and promote gut motility [20]. Lower metabolic rate is the key factor-induced hypothermia. The main factors that increases ghrelin secretion include fasting, nocturnal sleeping, lower plasma glucose level, lower-fat meal, anorexia, and lower body weight [21]. The factors that decrease ghrelin secretion include short bowel syndrome, higher growth hormone, and obese [15]. Brown adipose tissue is an important organ in energy expenditure and thermogenesis. Meanwhile, as an endocrine organ, IBA secreted fatty acids, triiodothyronine, leptin, adiponectin, resistin, and heat into blood circulation [22]. The effective signals of IBA on ghrelin secretion may be confirmed directly from the endocrine hormones and indirectly from immunological or neural system. A high-fat-diet decreases and a carbohydrate diet increases plasma ghrelin level [23]. Furthermore, the food intake response is amplified in rats fed with fat-diet, so that the more fat food intake induces indigestion. The plasma lipid levels are partly mechanisms in the inhibitory of ghrelin secretion [15]. Here, the persistent fat-diet elevated TP and TC levels, which may be related to the reverse of increased ghrelin expression and secretion. While TC in fat-diet is metabolized to free fatty acids, the uncoupling proteins are activated to increase thermogenesis. ALZ increases secretion of ghrelin, as an effective molecule for reliving the hypothermia with indigestion in rats with Spleen Yang Deficiency. The ghrelin O-acyltransferase is acylated at ghrelin-serine-3 with an octanoate to confer its biological activities [24]. However, it has not determined the exact molecules of ALZ to upregulate ghrelin secretion; meanwhile, some NEI network clues are not directly hinted from nutrients, hormones, and autonomic nervous system in certain physiological or pathological statuses [25, 26]. ALZ is the classic warming prescription in TCM. A series of researches about the warming prescriptions had found that uncoupling proteins are the major effectors in the pathway of nonshivering thermogenesis [27]. The uncoupling proteins would dissipate the proton gradient across the mitochondrial inner membrane, thereby, dissociating respiration from ATP synthesis [27, 28]. The mitochondrial free fatty acids are thought to be the key species activating uncoupling proteins [27]. ALZ could enhance the function of free fatty acids on uncoupling protein functions to induce thermogenesis [13].

There are three conclusions that could be made from these data. (1) A rat model of the hypothermia with indigestion had been replicated with IBA removal following a fat-diet. The adaptive thermogenesis, thyroid-related hormones, plasma metabolites, and their related behaviors were in accordance with the features of Spleen Yang Deficiency in TCM. (2) ALZ downregulated ghrelin-mRNA expression and promoted ghrelin secretion into blood, at least, leading to the decrease of catabolism for energy storage and the improvement of gut motility. Some data supported ghrelin as one of the effective bimolecular for ALZ pharmacological actions, such as adaptive thermogenesis, thyroid-related hormones, plasma metabolites, and the related behaviors. (3) A special network for drug-NEI disease should be set up as a dry model of the hypothermia with indigestion, so that the detail actions of ALZ could delicately be analyzed in the multilayer [18]. The role of ghrelin in ALZ therapy should be further confirmed in clinic for the patients suffering from the hypothermia with indigestion.

## Figures and Tables

**Table 1:** Growth ratio of weight in rats with IBA removed (mean ± SD, *n*_*i*_ = 18).

Groups	Δ Ratio
Control	0.71 ± 0.06
Model	0.45 ± 0.04^aa^
Fat-diet	0.38 ± 0.05^aabb^
ALZ	0.50 ± 0.03^aabbcc^

Versus Control, ^a^ *P* < 0.05, ^aa^ *P* < 0.01; versus Model, ^b^ *P* < 0.05, ^bb^ *P* < 0.01; versus Fat-diet, ^c^ *P* < 0.05, ^cc^ *P* < 0.01.

**Table 2:** Plasma metabolite levels in rats with IBA removed (mean ± SD, *n*_*i*_ = 18).

Groups	TP (g/L)	TC (mmol/L)	TG (mmol/L)	Glu (mmol/L)
Control	62.92 ± 3.24	1.85 ± 0.12	0.81 ± 0.08	10.66 ± 0.70
Model	56.36 ± 3.20^aa^	1.50 ± 0.16^aa^	0.40 ± 0.08^aa^	10.50 ± 0.76^aa^
Fat-diet	58.47 ± 2.42^aabb^	1.66 ± 0.13^aabb^	0.49 ± 0.07^aabb^	9.14 ± 0.95^aabb^
ALZ	54.55 ± 2.33^aabbcc^	2.27 ± 0.12^aabbcc^	0.53 ± 0.08^aabbcc^	12.16 ± 1.17^aabbcc^

Versus Control, ^a^ *P* < 0.05, ^aa^ *P* < 0.01; versus Model, ^b^ *P* < 0.05, ^bb^ *P* < 0.01; versus Fat-diet, ^c^ *P* < 0.05, ^cc^ *P* < 0.01.

**Table 3:** Plasma thyroid-related hormones in rats with IBAremoved (mean ± SD, *n*_*i*_ = 18).

Groups	*T*_3_ (ng/mL)	*T*_4_ (ng/mL)	TSH (*μ*IU/mL)
Control	0.82 ± 0.06	50.14 ± 7.85	2.52 ± 0.27
Model	0.89 ± 0.08^aa^	55.48 ± 10.47^aa^	2.20 ± 0.47^aa^
Fat-diet	0.77 ± 0.07^aabb^	44.51 ± 7.00^aabb^	2.74 ± 0.22^aabb^
ALZ	0.65 ± 0.03^aabbcc^	40.23 ± 6.89^aabbcc^	3.19 ± 0.12^aabbcc^

Versus Control, ^a^ *P* < 0.05, ^aa^ *P* < 0.01; versus Model, ^b^ *P* < 0.05, ^bb^ *P* < 0.01; versus Fat-diet, ^c^ *P* < 0.05, ^cc^ *P* < 0.01.

**Table 4:** Thermogenic peak (^°^C) in rats with IBA removed (mean ± SD, *n*_*i*_ = 18).

Groups	*d*_42_	*d*_70_	*d*_98_
Control	38.85 ± 0.66	39.00 ± 0.58	39.38 ± 0.57
Model	38.93 ± 0.86	38.64 ± 0.47^aa^	38.13 ± 0.43^aa^
Fat-diet	38.76 ± 0.61	38.38 ± 0.46^aabb^	38.53 ± 0.52^aabb^
ALZ	39.06 ± 0.83	38.50 ± 0.47^aabb^	38.70 ± 0.51^aabbcc^

Versus Control, ^a^ *P* < 0.05, ^aa^ *P* < 0.01; versus Model, ^b^ *P* < 0.05, ^bb^ *P* < 0.01; versus Fat-diet, ^*c*^ *P* < 0.05, ^cc^ *P* < 0.01.

**Table 5:** Thermogenic AUC (^°^C·h) in rats with IBA removed (mean ± SD, *n*_*i*_ = 18).

Groups	*d*_42_	*d*_70_	*d*_98_
Control	76.72 ± 1.76	77.66 ± 1.00	78.88 ± 1.11
Model	76.83 ± 1.61	77.18 ± 1.02^aa^	76.21 ± 0.91^aa^
Fat-diet	76.70 ± 1.03	76.69 ± 1.00^aabb^	76.67 ± 0.87^aabb^
ALZ	76.90 ± 1.50	76.57 ± 1.17^aabb^	77.19 ± 0.94^aabbcc^

Versus Control, ^a^ *P* < 0.05, ^aa^ *P* < 0.01; versus Model, ^b^ *P* < 0.05, ^bb^ *P* < 0.01; versus Fat-diet, ^c^ *P* < 0.05, ^cc^ *P* < 0.01.

**Table 6:** Ghrelin secretion in rats with IBA removed (mean ± SD).

Groups	Plasma level	Stomach level	IHC level	mRNA level	Protein level
	(pg/mL, *n*_*i*_ = 18)	(*μ*g/mg, *n*_*i*_ = 18)	(OD/V, *n*_*i*_ = 18)	(2^−ΔΔCT^, *n*_*i*_ = 7)	(OD/OD, *n*_*i*_ = 7)
Control	1091.34 ± 245.11	81.15 ± 11.05	0.2729 ± 0.0225	1.7154 ± 0.3963	0.7370 ± 0.1149
Model	2508.78 ± 298.22^aa^	29.44 ± 2.66^aa^	0.1793 ± 0.0207^aa^	6.2047 ± 2.2695^aa^	1.4357 ± 0.2766^aa^
Fat-diet	1366.40 ± 473.17^abb^	26.93 ± 4.18^aabb^	0.1930 ± 0.0071^aabb^	2.7509 ± 0.8356^aabb^	0.9169 ± 0.0749^aabb^
ALZ	1552.76 ± 460.08^aabbc^	39.19 ± 3.07^aabbcc ^	0.2112 ± 0.0137^aabbcc ^	0.4372 ± 0.1574^aabbcc^	0.2405 ± 0.1444^aabbcc^

Versus Control, ^a^ *P* < 0.05, ^aa^ *P* < 0.01; versus Model, ^b^ *P* < 0.05, ^bb^ *P* < 0.01; versus Fat-diet, ^c^ *P* < 0.05, ^cc^ *P* < 0.01.
